# Super-Mini Percutaneous Nephrolithotomy for Nephrolithiasis: A Systematic Review and Meta-Analysis

**DOI:** 10.7759/cureus.32253

**Published:** 2022-12-06

**Authors:** Mohamed Zeid, Hani Sayedin, Natrajan Sridharan, Arun Narayanaswamy, Fawzi Abul, Prem Thomas Jacob, Subhasis Giri, Kemal Sarica, Shabir Almousawi

**Affiliations:** 1 Urology, University Hospital Limerick, Limerick, IRL; 2 Urology, Warrington and Halton Teaching Hospitals NHS Foundation Trust, Warrington, GBR; 3 Urology, Vedanayagam Hospital, Coimbatore, IND; 4 Urology, Sabah Al-Ahmad Urology Centre, Kuwait, KWT; 5 Urology, Biruni University, Istanbul, TUR

**Keywords:** super-mini pcnl, pediatric urinary stone disease, stone surgery, urinary stones, mini-pcnl, pcnl

## Abstract

We aimed to conduct a systematic review and meta-analysis to summarize the current evidence regarding the role of super-mini percutaneous nephrolithotomy (SMP), which refers to a 7-Fr nephroscope placed through a tract sized 10-14 Fr, in treating renal stones and compare its outcomes with the standard mini-percutaneous nephrolithotomy (PCNL) techniques.

A systematic literature search was conducted on the Medline database via PubMed and SCOPUS until May 2022 to retrieve the relevant studies. The titles and abstracts of unique records were screened for eligibility, followed by the full-text screening of potentially eligible abstracts. Data extraction was performed by two independent reviewers. The risk of bias assessment was conducted based on the study design. Open Meta (Analyst) and Review Manager 5.4 were used to perform all analyses. A total of 14 studies (n = 4,323 patients) were included, with two randomized controlled trials, one single-arm trial, and 11 cohort studies.

The stone-free rate (SFR) of SMP was 91.4%. The pooled analysis showed no significant difference between SFR in mini-PCNL (mean difference (MD) = 1.03, 95% confidence interval (CI) = (0.99, 1.06), p = 0.12) and flexible ureteroscopy (MD = 0.84, 95% CI = (0.4, 1.76), p = 0.65]. On the other hand, SMP had a better SFR rate when compared with retrograde intrarenal surgery (MD = 1.3, 95% CI = (1.01, 1.66), p = 0.04). The pooled mean operative time of SMP was 49.44 minutes (95% CI = (41, 57.88), p < 0.001), which was longer than mini-PCNL (MD = 1.92, p < 0.001) and shorter than ureteroscopy (MD = -17.17, p < 0.00001). In the SMP group, the postoperative complications included fever (>38°C), pain, and hematuria, with an incidence of 7.6%, 2.3%, and 3.4%, respectively. The mean length of hospital stay after SMP was 2.4 days (95% CI = (2.17, 2.7), p < 0.001).

The current evidence suggests that SMP is a safe and effective technique in the management of renal stones in both children and adults.

## Introduction and background

Percutaneous nephrolithotomy (PCNL) is the most frequent method for treating large and complex renal stones, with reported stone clearance rates of up to 85% [1]. The main objective of PCNL is to eliminate stones while reducing morbidity and complications such as bleeding and pain. Complication rates have been linked to the size of the tract, according to previous studies [2]. The current minimally invasive modalities of PCNL, such as ultra-mini PCNL (a 16-F sheath) and micro-PCNL (a 4.85-Fr tract) [3], have greatly reduced the tract size [4]. Miniaturized PCNL still has concerns regarding fragments migrating to other regions of the pelvicalyceal system. Improved visualization, intrarenal pressure prevention, and stone fragment clearance are just a few of the numerous advantages of super-mini PCNL (SMP) over existing mini-PCNL techniques [5].

Recently, several studies were performed to compare the safety and efficacy of SMP with other mini-PCNL techniques [5-18]. Guddeti et al. performed a randomized clinical trial (RCT) to evaluate the efficacy of SMP versus standard PCNL in the management of renal calculi (<2 cm). Their findings showed that SMP was associated with longer operative time, less hemoglobin drop, lower pain score, less analgesic requirement, and shorter hospital stay. On the other hand, both groups were comparable in terms of stone-free rate [12]. In a single-center study, Simayi et al. examined the safety and efficacy of SMP versus other mini-PCNL techniques in upper urinary tract stones in children. The study concluded that SMP was more effective than standard mini-PCNL, and it was associated with a higher tubeless rate and shorter hospital stay [16]. In this study, we aimed to conduct a systematic review and meta-analysis to summarize the current evidence regarding the role of SMP in treating renal stones and compare its outcomes with the standard mini-PCNL techniques.

## Review

Methodology

This systematic review and meta-analysis was conducted in line with the recommendations of the Cochrane Collaborative Group [19] and the Preferred Reporting Items for Systematic Reviews and Meta-Analyses checklist [20].

Literature Search and Eligibility Criteria

We performed a systematic literature search on the Medline database via PubMed and SCOPUS until May 2022 to retrieve studies that fulfilled the following criteria: studies that included adult patients (>18 years) with renal stones, regardless of their size; studies that assessed the intra and postoperative outcomes of SMP; studies that compared the outcomes of SMP with no intervention or other surgical modalities; and studies that were either single-arm, RCTs, or retrospective or prospective cohorts. We excluded studies with no reliable data for extraction regarding the intra and postoperative outcomes, non-original reports, and theses. We used a combination of the following queries to complete the bibliographic search: “renal stones.” “nephrolithotomy,” “nephrolithiasis,” “percutaneous nephrolithotomy,” and “super-mini percutaneous nephrolithotomy.” We included prospective and retrospective studies that were published in the English language and followed up with patients with AML through active surveillance. The search was not limited to a specific publication period, country, or language.

Screening and Data Extraction

Unique records were retrieved through the Endnote X8 program (Thomson Reuter, USA). The titles and abstracts of the unique records were screened for eligibility, followed by the full-text screening of potentially eligible abstracts. Both steps were performed by two independent reviewers, and any discrepancies were resolved by consensus. The following data from eligible studies were extracted by two reviewers: characteristics of the study’s objectives and design, main findings, population characteristics, preoperative data, intraoperative characteristics, follow-up duration, and postoperative outcomes. The risk of bias assessment of individual studies in this systematic review depended on the study design. RCTs were assessed using the Cochrane Risk-of-Bias tool for randomized trials (RoB 2). On the other hand, the risk of bias of non-randomized trials was assessed using the Newcastle-Ottawa Scale (NOS).

Statistical Analysis

The mean operative time, overall SFR, tubeless rate, postoperative complications, and the mean postoperative hospital stay for SMP were calculated based on one-arm analysis using the Open Meta (Analyst) software. Regarding the comparison between SMP and other modalities, we used Review Manager 5.4 software to generate the forest plot of the pooled analysis of each outcome. The mean difference (MD) between the studied groups based on the inverse variance (IV) model was calculated in terms of the Visual Analog Scale (VAS) score, postoperative hospital stay, and hemoglobin drop. On the other hand, to highlight the safety of SMP versus other modalities, we calculated the risk ratio (RR) based on the Mantel-Haenszel (M-H) model in terms of the complication rate, requiring auxiliary procedures, SFR at one and three months, tubeless rate, and Clavien-Dindo grades. Using the I^2^ statistic, we calculated the percentage of heterogeneity and inconsistency between studies, with values of 25%, 50%, and 75% deemed low, moderate, and high, respectively. The random-effect model was employed if the heterogeneity was considerable and I^2^ >50%; otherwise, the fixed-effect model was utilized. Subgroup analysis based on the arms of comparison was performed to minimize the risk of inconsistency.

Results

**Figure 1:**
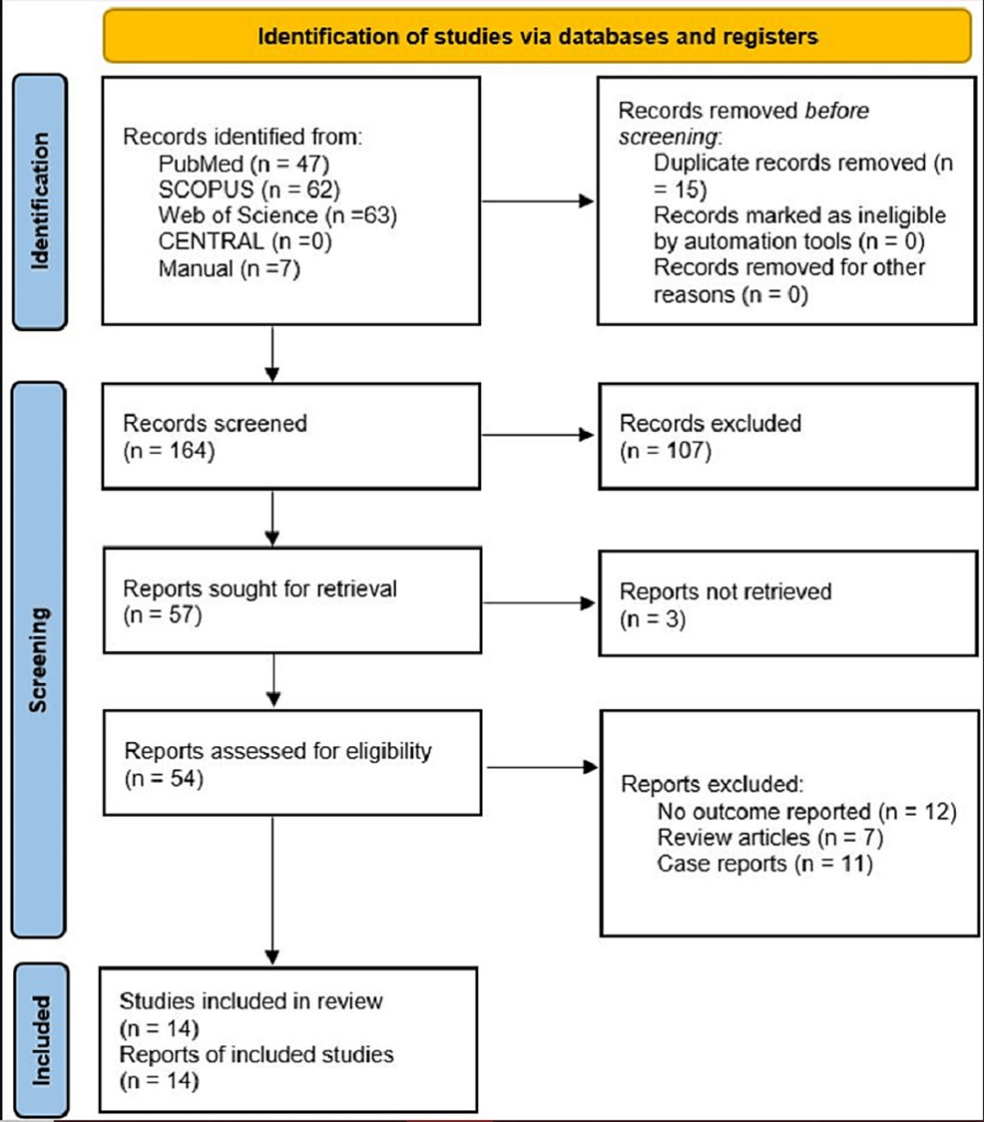
Identification of the included studies.

Our search approach yielded 181 studies, of which 106 were excluded based on titles and abstracts. Our meta-analysis finally included 14 studies that fulfilled the inclusion criteria, as shown in Figure 1.

Study Characteristics and Quality

We included two RCTs [7,12], one single-arm trial [5], and 11 cohort studies assessing SMP either alone or compared with different modalities [6,8-11,13-18]. Table 1 and Table 2 exhibit the main summary and baseline characteristics of included studies.

**Table 1:** Summary of the included studies. SMP = super-mini percutaneous nephrolithotomy; RIRS = retrograde intrarenal surgery; LPC = lower pole renal calculi; SFR = stone-free rate; sPCNL = standard percutaneous nephrolithotomy; F-URS = flexible ureterorenoscopy

Authors	Study design	Study arms	Inclusion criteria	Conclusions
Zeng et al. 2015 [5]	Prospective single-arm clinical trial	SMP	Patients with kidney stones <2.5 cm	SMP was a safe and efficient therapy for renal stones less than 2.5 cm in diameter. Patients with lower pole stones and stones not susceptible to retrograde intrarenal surgery may benefit from SMP
Cai et al. 2018 [6]	Retrospective cohort, single-arm	SMP	Patients with renal stones ≥20 mm	For the treatment of 20 mm renal stones, new SMP may be a safe and effective option. The new approach may allow SMP to be used to treat big kidney stones
Zeng et al. 2018 [7]	Randomized controlled trial	SMP versus RIRS	Patients with 1–2 cm LPC	SMP was more successful than RIRS for treating 1–2 cm LPC in SFR and auxiliary procedure rate. The problems and length of hospital stay were similar. The advantage of RIRS is that it causes less postoperative discomfort
Liu et al. 2018 [8]	Prospective cohort, double-arm	SMP versus mini-PCNL	Patients with single or more kidney stones measuring between 2 and 4 cm in diameter	In treating mild renal calculi, SMP was as successful as mini-PCNL, although it had a substantial advantage in terms of hospital stay and tubeless rate
Yuan et al. 2019 [9]	Retrospective cohort, double-arm	SMP versus mini-PCNL	Pediatric patients diagnosed with kidney stones	SMP seemed to be a safer therapy for children with kidney stones than mini-PCNL, with a decreased rate of postoperative sequelae
Fan et al. 2019 [10]	Retrospective cohort, single-arm	SMP	Patients who underwent SMP to treat the symptomatic LPSs after the failure of SWL or RIRS	SMP was a safe and effective supplemental option for symptomatic LPSs following the failure of SWL or RIRS, and it may even be a substitute for SWL or RIRS
Simayi et al. 2019 [11]	Retrospective cohort, single-arm	SMP	Patients with upper urinary tract stone diameter <3.5 cm	Ultrasonography-guided SMP was a safe and effective treatment option for moderate-sized upper urinary tract stones, with the benefit of avoiding radiation exposure, particularly beneficial for pediatric stone patients
Guddet et al. 2020 [12]	Randomized controlled trial	sPCNL versus SMP	Patients presenting with renal calculi <2 cm	SMP was as successful as sPCNL in treating renal calculi under 2 cm, but it was safer. Although SMP required more time in the operating room, it decreased the risk of bleeding and postoperative discomfort and a shorter hospital stay
Liu et al. 2020 [13]	Retrospective cohort study, double-arm	SMP versus mini-PCNL	Patients with a single or multiple renal stones >2 cm	According to the findings, SMP is an excellent treatment choice for stones under 4 cm and is more effective for stones measuring between 2 and 3 cm, reducing postoperative fever, blood loss, and discomfort
Xu et al. 2020 [14]	Retrospective cohort, double-arm	SMP versus F-URS	Obese patients with 20–30 mm renal stones	Both SMP and F-URS were equally effective in obese individuals with 20–30 mm renal stones. On the other hand, F-URS had a reduced complication rate, but SMP was superior in surgery duration, tubeless rate, stage two procedures, and total expenditures
Jia et al. 2021 [15]	Retrospective cohort, double-arm	SMP versus RIRS	Children with upper urinary tract calculus (1–2 cm)	In children with upper urinary tract calculus (1–2 cm), SMP was more successful than RIRS in achieving a higher SFR, lower re-treatment rate, and lower complication rate
Simayi et al. 2021 [16]	Retrospective cohort, double-arm	SMP	Children with upper urinary tract stones	SMP was more successful than mini-PCNL in treating pediatric middle-sized upper urinary tract stones, and it had a shorter length of stay and a greater tubeless rate
Pillai et al. 2022 [17]	Retrospective, cohort, double-arm	SMP versus RIRS	Patients with a single renal stone with a maximum diameter of 2 cm	In comparison to RIRS, SMP had considerably shorter surgical times, complication rates, hospital stays, and greater SFRs. However, SMP was linked to higher early postoperative discomfort
Yuan et al. 2022 [18]	Retrospective cohort, double-arm	SMP versus ureteroscopy	Patients with kidney stones diameter <2 cm	In the case of kidney stones, SMP was more effective than ureteroscopy. Infectious stones, preoperative urinary tract infection, preoperative blood glucose level, and positive urine culture Infection were unrelated to this

**Table 2:** Baseline characteristics of the included studies. URS = semi-rigid ureteroscopy; SMP = super-mini percutaneous nephrolithotomy; PCNL = percutaneous nephrolithotomy; RIRS = retrograde intrarenal surgery; F-URS = flexible ureterorenoscopy

Authors	Study arms	Sample	Age (years), mean ± SD	Sex, M/F	BMI, (kg/cm^2^) Mean ± SD	Stone size (mm), mean ± SD	Laterality, R/L	Comorbidities, yes (%)	Number of stones, single/multiple
Zeng et al. 2015 [5]	SMP	141	-	91/50	21.6 ± 4.5	22 ± 6	-	-	16/135
Cai et al. 2018 [6]	SMP	188	47.14 ± 15.13	115/73	23.76 ± 3.79	31.57±9.8	95/93	50 (26.6)	61/127
Zeng et al. 2018 [7]	SMP	80	49.4 ± 12.8	50/30	24.6 ± 4.1	15 ± 2.9	42/38	36 (45%)	-
	RIRS	80	47.1 ± 13.9	46/34	24.1 ± 3	14.3±3.4	38/42	23 (28.8%)	-
Liu et al. 2018 [8]	SMP	73	46.5 ± 14.4	51/23	24.2 ± 3.7	31 ± 11	-	27 (37%)	24/36
	Mini-PCNL	73	48 ± 11.4	41/32	24.7 ± 3.6	32 ± 7	-	25 (34.2%)	29/32
Yuan et al. 2019 [9]	Mini-PCNL	22	9.2 ± 3.75	15/7	-	-	13/9	-	-
	SMP	17	7.8±3.5	6/11	-	-	6/7	-	-
Fan et al. 2019 [10]	SMP	44	49.1 ± 13.7	26/18	23.8 ± 3.4	18.4 ± 6	19/25	-	39/5
Simayi et al. 2019 [11]	SMP	104	26.1 ± 23	64/40	20.9 ± 5.1	17.3 ± 0.6	-	-	65/39
Guddet et al. 2020 [12]	SMP	75	46.53 (20–80)^a^	51/24	26.02^b^	14.9 ± 7.3	43/32	34 (45%)	-
	sPCNL	75	48.36 (19–76)^a^	57/18	25.23^b^	14.8 ± 7.8	38/37	32 (43%)	-
Liu et al. 2020 [13]	SMP	1,380	48.4 ± 13.7	891/489	25.1 ± 14.5	32 ± 9.1	-	403 (29.2%)	381/999
	Mini-PCNL	1,380	48.7 ± 11.7	871/509	24.5 ± 10.5	32.2 ± 7.7	-	414 (30%)	386/994
Xu et al. 2020 [14]	F-URS	104	48.72 ± 13.56	71/33	34.09 ± 2.2	-	47/57	-	54/50
	SMP	48	49.96 ± 12.86	34/14	33.11 ± 2.17	-	21/27	-	19/29
Jia et al. 2021 [15]	SMP	36	4.5 ± 2.7	26/10	16.63 ± 2.6	14.18 ± 3	22/14	-	26/10
	RIRS	25	4.3 ± 2.5	15/10	15.73 ± 2.1	14 ± 2.8	15/10	-	18/7
Simayi et al. 2021 [16]	SMP	66	5 ± 2.3	41/25	-	20 ± 8	-	-	45/21
	Mini-PCNL	67	3.6 ± 3.5	34/33	-	15 ± 5.3	-	-	58/9
Pillai et al. 2022 [17]	SMP	75	48.36 (19–76)^a^	57/18	25.23^b^	14.8 ± 7.8	38/37	32 (43%)	-
	RIRS	74	48.56 (23–76)	51/23	25.62^b^	14.18 ± 3	35/39	43 (58%)	-
Yuan et al. 2022 [18]	SMP	10	-	70/34	-	-	-	64 (61.5%)	-
	URS	76	-	46/30	-	-	-	41 (54%)	-

The risk of bias of the two RCTs was high according to the second version of the Cochrane tool. The only single-arm trial was fair in quality following the National Institutes of Health quality assessment tool for before-after studies with no control group. The remaining 11 cohort studies were assessed by the NOS quality assessment tool for observational cohort studies. The quality of only two studies was poor, while the rest nine studies were of high quality.

Single-Arm Meta-Analysis for Super-Mini Percutaneous Nephrolithotomy Alone

**Figure 2:**
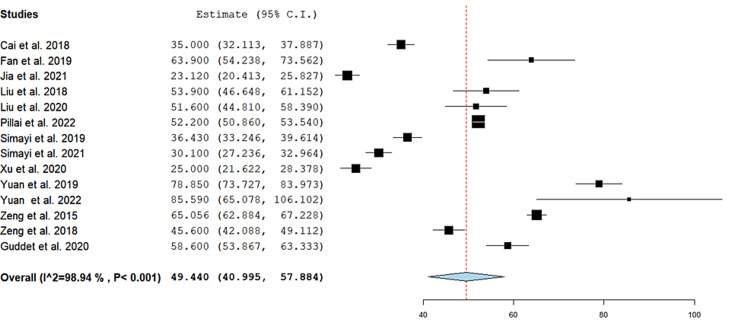
Average operation time of the included studies. Forest plot of the single-arm pooled analysis of operative time in patients who underwent super-mini percutaneous nephrolithotomy [5-18]. 95% CI = 95% confidence interval

Operative time: The pooled mean operation time was 49.44 minutes (95% CI = (41, 57.88), p < 0.001). The results were heterogeneous (I^2^ = 98.9%, p < 0.001), and the heterogeneity could not be resolved (Figure 2).

Operation tubes: Regarding the tubeless rate, the total tubeless, JJ stent rate, nephrostomy tube, and ureteral catheter, the following percentages were reported: 91.3% (95% CI = (84.6, 98.1), p < 0.001), 51.4% (95% CI = (36, 66.9), p < 0.001), 6.6% (95% CI = (24.6, 48.6), p < 0.001), 11% (95% CI = (0.05, 21.6), p = 0.041), and 2.9% (95% CI = (1.4, 4.3), p < 0.001), respectively. The pooled results were heterogenous for all except ureteral catheters (I^2^ = 97.87%, p < 0.001), (I^2^ = 98.17%, p < 0.001), (I^2^ = 96.44%, p < 0.001), (I^2^ = 96.64%, p < 0.001), and (I^2^ = 0, p = 0.170), respectively.

**Figure 3:**
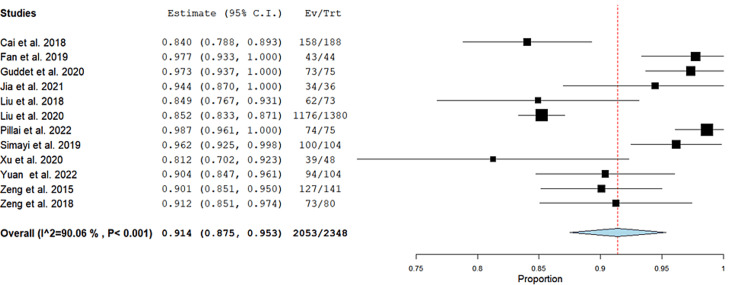
Initial after-operation stone-free rate of the included studies. Forest plot of the single-arm pooled analysis of stone-free rate in patients who underwent super-mini percutaneous nephrolithotomy [5-15,17]. 95% CI = 95% confidence interval

Stone-free rate (%), (initial after operation): The incidence SFR was 91.4% (95% CI = (87.5, 95.3), p < 0.001). The pooled results were heterogeneous (I^2^ = 90.1%, p < 0.001), and the heterogeneity could not be resolved (Figure 3).

Postoperative complications: Regarding postoperative complications including fever (>38°C), pain, and haematuria, the incidence was 7.6% (95% CI = (6.5, 8.8), p < 0.001), 2.3% (95% CI = (0.7, 3.9), p = 0.006), and 3.4% (95% CI = (1.1, 5.7), p = 0.004), respectively. The pooled results were heterogenous for all except fever (I^2^ = 0, p = 0.103), (I^2^ = 56%, p < 0.057), and (I^2^ = 75.85%, p = 0.002), respectively. The heterogeneity of pain and haematuria were resolved by excluding Liu et al. (I^2^ = 0, p = 0.525) and (I^2^ = 0, p < 0.720), respectively, and the incidence became 1.3% (95% CI = (-0.1, 2.7), p = 0.061) for pain and 2.1% (95% CI = (0.6, 3.5), p = 0.005) for hematuria.

**Figure 4:**
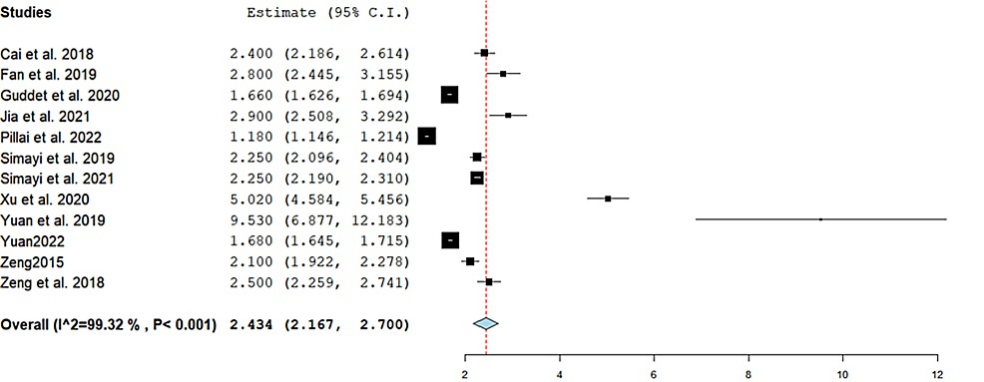
Average postoperative hospital stay. Forest plot of the single-arm pooled analysis of postoperative hospital stay in patients who underwent super-mini percutaneous nephrolithotomy [5-7,9-12,14-18]. 95% CI = 95% confidence interval

Postoperative hospital stay (days): The mean length of hospital stay was 2.4 (95% CI = (2.17, 2.7), p < 0.001). The pooled results were heterogeneous (I^2^ = 99%, p = <0.001), and this heterogeneity could not be resolved (Figure 4).

The rest of the postoperative outcomes are presented in Table 3.

**Table 3:** Summary of the outcome. SMP = super-mini percutaneous nephrolithotomy; PCNL = percutaneous nephrolithotomy; RIRS = retrograde intrarenal surgery; 95% CI = 95% confidence interval; RR = risk ratio; MD = mean difference

	Outcome or subgroup	Studies	Participants	Effect estimates		
				RR or MD	95% CI lower limit	95% CI upper limit
1	Hematuria	4		Subtotals only		
1.1	SMP vs. mini-PCNL	2	2,906	0.86	0.65	1.15
1.2	SMP vs. sPCNL	1	150	5	0.24	102.42
1.3	SMP vs. RIRS	1	160	3	0.32	28.23
2	Pain (SMP vs. mini-PCNL)	2	2,906	0.81	0.56	1.16
3	Fever (>38°C)	6		Subtotals only		
3.1	SMP vs. sPCNL	1	150	5	0.6	41.78
3.2	SMP vs. RIRS	2	221	0.37	0.14	0.98
3.3	SMP vs. mini-PCNL	3	2,945	0.72	0.57	0.89
4	DJ stent	4		Subtotals only		
4.1	SMP vs. mini-PCNL	3	2,945	0.96	0.87	1.05
4.2	SMP vs. ureteroscopy	1	152	1.41	1.18	1.7
5	Nephrostomy tube (SMP vs. mini-PCNL)	2	2,906	0.46	0.21	1
6	Total tubeless	5		Subtotals only		
6.1	SMP vs. mini-PCNL	3	3,039	108.6	6.84	1723.07
6.2	SMP vs. sPCNL	1	150	0.3	0.14	0.61
6.3	SMP vs. RIRS	1	149	4.6	2.03	10.47
7	Tubeless rate	5		Subtotals only		
7.1	SMP vs. Mini-PCNL	3	3,039	1.46	1.04	2.03
7.2	SMP vs. sPCNL	1	150	1	0.97	1.03
7.3	SMP vs. RIRS	1	149	1	0.97	1.03
8	Clavien–Dindo grade I	6		Subtotals only		
8.1	SMP vs. RIRS	2	309	1	0.2	4.85
8.2	SMP vs. mini-PCNL	5	3,039	0.78	0.67	0.9
8.3	SMP vs. ureteroscopy	1	152	5.2	1.94	13.93
9	Clavien–Dindo grade II	5		Subtotals only		
9.1	SMP vs. RIRS	2	210	0.18	0.05	0.67
9.2	SMP vs. mini-PCNL	2	2,906	0.26	0.02	3.24
9.3	SMP vs. ureteroscopy	1	152	3.71	1.56	8.84
10	Clavien–Dindo grade III (SMP vs. RIRS)	4		Subtotals only		
10.1	SMP vs. RIRS	1	149	0.2	0.01	4.04
10.2	SMP vs. mini-PCNL	2	2,906	0.69	0.5	0.96
10.3	SMP vs. ureteroscopy	1	152	15	0.79	284.8
11	Visual analog score-24 hours	3		Subtotals only		
11.1	SMP vs. sPCNL	1	150	0.45	0.29	0.61
11.2	SMP vs. RIRS	1	149	-0.7	-0.9	-0.5
11.3	SMP vs. mini-PCNL	1	2,760	-0.2	-0.27	-0.13
12	Visual analog score-12 hours	2		Subtotals only		
12.1	SMP vs. sPCNL	1	150	0.72	0.55	0.89
12.2	SMP vs. RIRS	1	160	0.7	0.2	1.2
13	Visual analog score-6 hours	4				
13.1	SMP vs. sPCNL	1	150	1.34	1.17	1.51
13.2	SMP vs. mini-PCNL	1	2,760	-0.4	-0.51	-0.29
13.3	SMP vs. RIRS	2	309	0.6	0.41	0.79
14	SFR, %, three months	3		Subtotals only		
14.1	SMP vs. mini-PCNL	1	2,760	0.99	0.97	1.01
14.2	SMP vs. ureteroscopy	1	152	1.08	0.98	1.2
14.3	SMP vs. RIRS	1	160	1.14	1.01	1.2
15	SFR, %, one month	4		Subtotals only		
15.1	SMP vs. RIRS	2	150	0.09	0.01	1.6
15.2	SMP vs. mini-PCNL	1	2,906	0.75	0.6	0.9
16	Postoperative hospital stay, days	7		Subtotals only		
16.1	SMP vs sPCNL	1	150	0.48	0.43	0.5
16.2	SMP vs. ureteroscopy	2	332	0.56	-1.67	2
16.3	SMP vs. mini-PCNL	1	39	-1.09	-4.48	2
16.4	SMP vs. RIRS	3	370	-0.51	-1.33	0
17	Hemoglobin drop, g/dL	3		Subtotals only		
17.1	SMP vs. sPCNL	1	150	4.5	2.66	6.3
17.2	SMP vs. PIRS	2	309	1.78	-6.15	9

Double-Arm Meta-Analysis

**Figure 5:**
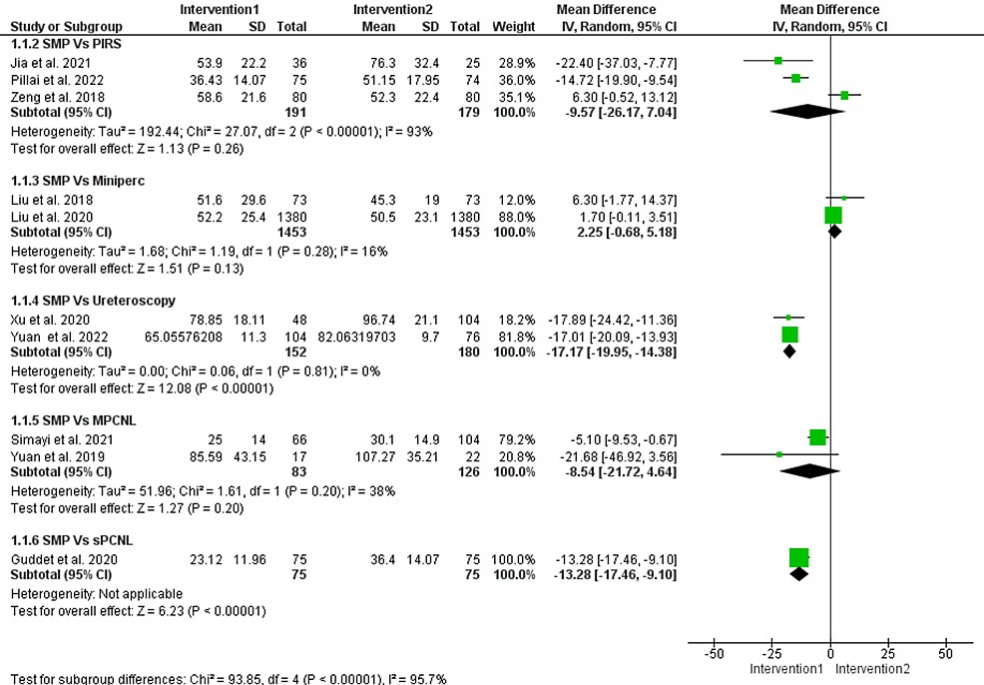
Operative time of the included studies. Forest plot of the pooled analysis of operative time in patients who underwent SMP versus RIRS [7,15,17], mini-PCNL [8,9,11,13], ureteroscopy [14,18], and sPCNL [12]. SMP = super-mini percutaneous nephrolithotomy; mini-PCNL = mini-percutaneous nephrolithotomy; sPCNL = standard percutaneous nephrolithotomy; RIPS = retrograde intrarenal surgery; 95% CI = 95% confidence interval; IV = inverse variance

Operative time: The operation time of SMP was longer than mini-PCNL (MD = 1.92, 95% CI = (0.15, 3.69), p < 0.001), and the data were homogenous (I^2^ = 16%, p = 0.28). On the other hand, the operation time of SMP was shorter than ureteroscopy (MD = -17.17, 95% CI = (-19.95, -14.38), p < 0.00001), and the pooled results were homogenous (I^2^ = 0, p = 0.81). There was no difference between SMP and RIRS (MD = -9.57, 95% CI (-26.17, 7.04), p = 0.26). The data were heterogenous (I^2^ = 93%, p < 0.00001), and this heterogeneity was resolved by excluding Zeng et al. (I^2^ = 0, p = 0.33). The homogenous results showed that SMP had a shorter duration than RIRS (MD = -15.58, 95% CI (-20.46, -10.69), p < 0.00001) (Figure 5).

**Figure 6:**
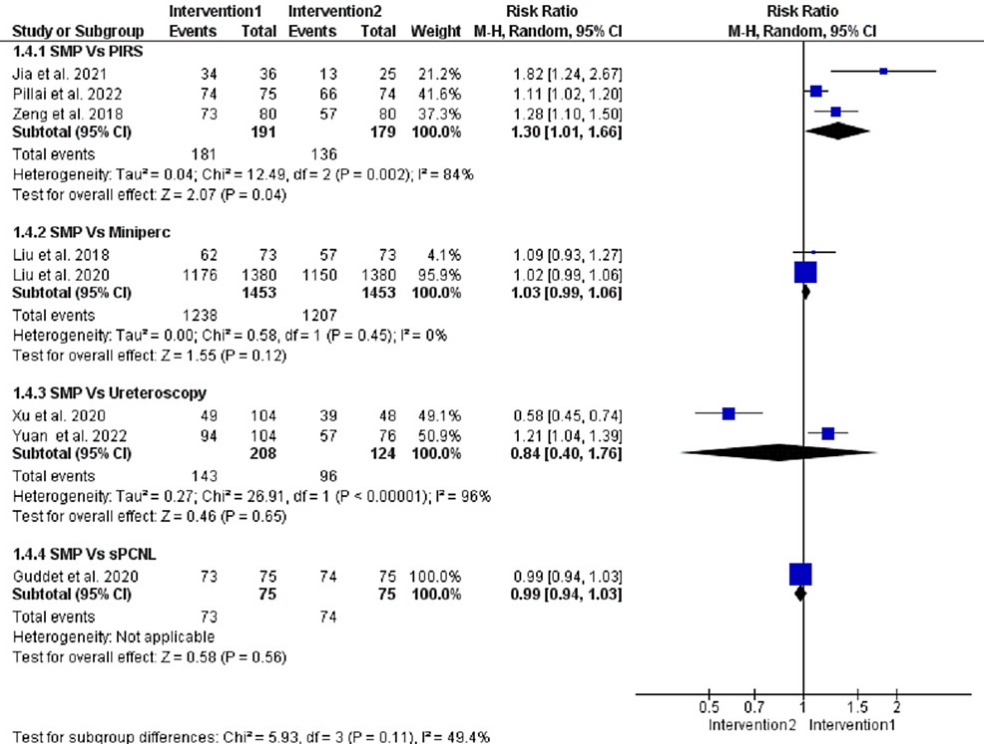
Stone-free rate (%). Forest plot of the pooled analysis of stone-free rate in patients who underwent SMP versus RIRS [7,15,17], mini-PCNL [8,13], ureteroscopy [9,14], and sPCNL [12]. SMP = super-mini percutaneous nephrolithotomy; mini-PCNL = mini-percutaneous nephrolithotomy; sPCNL = standard percutaneous nephrolithotomy; RIRS = retrograde intrarenal surgery; 95% CI = 95% confidence interval; M-H = Mantel-Haenszel

Stone-free rate (%), (initial after operation): The pooled analysis showed no significant results between SFR and mini-PCNL (MD = 1.03, 95% CI = (0.99, 1.06), p = 0.12], and the data were homogenous (I^2^ = 0, p = 0.45). Moreover, there were no significant results between SMP and ureteroscopy (MD = 0.84, 95% CI = (0.4, 1.76), p = 0.65]. However, the data were heterogenous (I^2^ = 96%, p = <0.00001), and this heterogeneity could not be resolved. On the other hand, SMP had a better SFR when compared with RIRS (MD = 1.3, 95% CI = (1.01, 1.66), p = 0.04). However, the data were heterogenous (I^2^ = 84%, p = 0.002), and this heterogeneity could not be resolved (Figure 6).

The rest of the intraoperative and postoperative outcomes are shown in Table 3.

Discussion

In this systematic review and meta-analysis, we included 14 studies (n = 4,323 patients) to assess the safety and efficacy of SMP in both children and adults, either alone or compared with different modalities. The SFR of SMP was 91.4%, while the pooled analysis showed no significant difference between SFR and mini-PCNL (MD = 1.03, 95% CI = (0.99, 1.06), p = 0.12). Moreover, there were no significant differences between SMP and ureteroscopy (MD = 0.84, 95% CI = (0.4, 1.76), p = 0.65]. On the other hand, SMP had a better SFR when compared with RIRS [MD = 1.3, 95% CI = (1.01, 1.66), p = 0.04). Despite the recent rise in popularity of mini-PCNL techniques, none have been able to achieve the same SFR as standard PCNL. Thus, this finding is substantial and demonstrates the significance of SMP for percutaneous stone management [12].

Dr. Peter Alken, one of the founding fathers of PCNL, believes that the growing interest in PCNL is due to various factors. He finds that the SMP system has solved the shortcomings of standard mini-PCNL systems, such as effective stone removal, sufficient irrigation, and increased endoscopic vision [21]. He concluded that “based on my more than 40 years of experience with percutaneous stone removal and intense knowledge of the changes that were introduced I think it is justified to state the SMP technique is the most significant progress in this field, and it will likely become the dominant method for percutaneous stone management in the future.” SFRs were from 60% to 90% when PCNL was miniaturized using ultra-mini PCNL (UMP), mini-perc, and micro-perc. Compared to previous minimally invasive PCNL investigations, the SMP group had a higher SFR [3,18,21]. Negative pressure suction is used to remove stone fragments during the SMP treatment, with the objective of leaving the patient stone-free at the end of the surgery [22,23]. Surgeons are able to easily fragment the stones with the clear vision provided by the simultaneous extraction of fragments using suction. The 3-Fr grasper may also be used to remove stone fragments from the pelvicalyceal system [7].

The pooled mean operative time of SMP was 49.44 minutes (95% CI = (41, 57.88), p < 0.001]. The operation time of SMP was longer than mini-PCNL (MD = 1.92, 95% CI = (0.15, 3.69), p < 0.001] and shorter than ureteroscopy (MD = -17.17, 95% CI = (-19.95, -14.38), p < 0.001]. There was no difference between SMP and both RIRS and mini-PCNL (MD = -9.57, 95% CI = (-26.17, 7.04), p = 0.26]. One explanation for the observed discrepancy was the additional time needed in the SMP group for laser fragmentation and dusting. In comparison to RIRS, larger fragments may immediately pass through the oblique side-port, saving time on stone fragmentation. At the same time, increasing the Holmium laser energy speeds up the lithotripsy process. In PCNL, Jia et al. prefer Holmium laser lithotripsy [15]. A 10- to 14-F access sheath with a 7-F nephroscopy and a suction-evacuation function with increased irrigation were introduced by Zeng et al. [5]. The unique suction-evacuation mechanism of SMP increased fragment removal efficiency while lowering intrapelvic pressure. The first-generation SMP was demonstrated to be a safe and successful therapy for renal stones less than 2.5 cm in diameter [5]. For lower pole stones less than 2 cm in diameter, the first-generation SMP was compared to RIRS. Although SMP had a greater SFR and auxiliary rate, it was shown to be associated with more postoperative pain than other methods [12]. For pediatric renal stones, the first-generation SMP was also a safe therapeutic option that had good effectiveness, shorter surgical time, and less general anesthetic required [24]. Although the first-generation SMP was a unique and effective method, it had two significant problems. One reason for this is that the access sheath of the SMP was composed of a clear plastic material, which made it more bendable. The acute angle made access to the calyx difficult for the plastic sheath. Using the negative pressure method, it is possible that irrigation fluid from the nephroscopy may drive fragments back into the collecting system, reducing the effectiveness of fragment extraction. Therefore, Cia et al. updated the SMP’s access sheath mechanism to address these two issues by employing a metal straight sheath and a handle [6]. As an irrigation channel, the straight sheath featured a two-layered metal construction. The distal tip of the straight sheath had side holes that enabled irrigation to exit. One-direction suction enhanced fragment evacuation without nephroscopy irrigation.

The postoperative complications included fever (>38°C), pain, and hematuria, with an incidence of 7.6%, 2.3%, and 3.4%, respectively. The mean length of hospital stay was 2.4 days (95% CI = (2.17, 2.7), p < 0.001). Therefore, many authors have suggested the use of SMP as an initial option for treating renal stones. According to the findings of Fan et al., SMP is a viable alternative to SWL or RIRS in patients with symptomatic lower pole renal stones (LPSs). The low complication rate and high SFR of SMP make it an excellent first-line treatment for patients with LPSs [17]. Cia et al. claimed that when they reduced the size of the access sheath to reduce the likelihood of serious bleeding, the findings indicated that no transfusion and arterial embolization were necessary. In addition, patients who got New-SMP therapy were discharged from the hospital after a 2.4-day stay [6]. The high tubeless rate of 87.2% at New-SMP may have been a contributing factor. It was shown that smaller nephrostomy tracts resulted in less bleeding, a shorter hospital stay, and a higher tubeless success rate. Most urologists, however, are worried about the increased risk of urosepsis and high renal pelvic pressure that come with employing short tracts [7]. However, our findings showed that the incidence of postoperative fever was low (7.6%). Patients who received RIRS instead of PCNL for the treatment of kidney stones spent considerably less time in the hospital, according to a recently published meta-analysis. However, in patients who were treated tubeless after PCNL, the length of stay in the hospital was dramatically shortened [25,26].

We acknowledge that our study has some limitations, including the small sample size of included studies, the significant unresolved heterogeneity, and the inability to conduct subgroup analysis or risk of publication analysis based on the Egger test due to the lack of relevant data and the small number of included studies.

## Conclusions

The current evidence suggests that SMP is a safe and effective technique in the management of renal stones in both children and adults. SMP, in addition to its low traumatic effect, showed a high SFR, low complication rate, and rapid recovery after the operation. Further RCTs are required to validate these findings and highlight the role of other factors such as the learning curve, patient’s age, and stone-related factors, including location, size, and complexity on the SFR.
